# Sink-Type-Dependent Data-Gathering Frameworks in Wireless Sensor Networks: A Comparative Study

**DOI:** 10.3390/s21082829

**Published:** 2021-04-16

**Authors:** Rezoan Ahmed Nazib, Sangman Moh

**Affiliations:** Department of Computer Engineering, Chosun University, 309 Pilmun-daero, Dong-gu, Gwangju 61452, Korea; rezoan.nazib@chosun.kr

**Keywords:** wireless sensor network, static sink, mobile sink, energy efficiency, routing protocol, data gathering, aerial sink, unmanned aerial vehicle, base station

## Abstract

Owing to automation trends, research on wireless sensor networks (WSNs) has become prevalent. In addition to static sinks, ground and aerial mobile sinks have become popular for data gathering because of the implementation of WSNs in hard-to-reach or infrastructure-less areas. Consequently, several data-gathering mechanisms in WSNs have been investigated, and the sink type plays a major role in energy consumption and other quality of service parameters, such as packet delivery ratio, delay, and throughput. However, the data-gathering schemes based on different sink types in WSNs have not been investigated previously. This paper reviews such data-gathering frameworks based on three different types of sinks (i.e., static, ground mobile, and aerial mobile sinks), analyzing the data-gathering frameworks both qualitatively and quantitatively. First, we examine the frameworks by discussing their working principles, advantages, and limitations, followed by a qualitative comparative study based on their main ideas, optimization criteria, and performance evaluation parameters. Next, we present a simulation-based quantitative comparison of three representative data-gathering schemes, one from each category. Simulation results are shown in terms of energy efficiency, number of dead nodes, number of exchanged control packets, and packet drop ratio. Finally, lessons learned from the investigation and recommendations made are summarized.

## 1. Introduction

Wireless sensor networks (WSNs) are among the most popular research topics among researchers in the field of wireless networking [1]. A WSN comprises multiple wirelessly interconnected multifunctional devices—with limited energy capabilities—called sensor nodes. The sensor nodes can be mobile or stationary and are placed in a region of interest (ROI) with at least one sink node [2]. The combination of a large number of sensor nodes in a WSN makes it ideal for a wide variety of applications [3], including area monitoring [4], health care monitoring [5], environmental observations [6], air quality monitoring [7], forest fire detection [8,9], landslide detection [10], water quality monitoring [11], natural disaster prevention [12], industrial monitoring [13], machine condition monitoring [14], data logging, waste monitoring [15], border surveillance [16], oil pipe monitoring [17], and scientific observations [18,19]. With the demand for automation and remote surveillance systems, WSN-based applications are increasing rapidly [20]. The increasing number of IoT devices and sensors generates huge amounts of data [21]. As sensor nodes are inexpensive, usually, they are used for single time deployment [22], especially in remote or hard-to-reach areas. To achieve the maximum potential of WSNs, it is important to ensure that the WSN data-gathering process is designed to conserve energy and increase the lifetime of the network [23].

Conventional WSN data-gathering schemes involve sensor nodes and sinks. A sink node is equipped with high energy and processing capabilities and is responsible for gathering all the data transmitted from the sensor nodes [24]. The sensor nodes disseminate data in a multi-hop or single-hop fashion. Compared to multi-hop methods, the single-hop approach consumes more energy [25] for a longer transmission distance. Moreover, the data generated from neighboring sensors are often redundant and correlated. Consequently, different multi-hop methods for data gathering have been examined in the literature such as clustering [26,27], tree-based [28], and hybrid techniques [29]. Clustering techniques have gained more acceptance in the research community for achieving network scalability, and several such techniques have been reported in the literature [30].

In multi-hop data transmission in WSNs, where the sink node is static, the sensor nodes close to it are more likely to run out of energy in comparison to other nodes because of the concentration of data traffic toward the sink; this is also known as the hotspot problem [31]. To solve this problem, a mobile sink has been used—the hotspot around the sink changes with the mobility of the sink—to provide load balancing in WSNs [32]. Mobile sink-based data gathering is relatively new compared to traditional data-gathering schemes using static sinks [33]. The mobile sink is responsible for entering a ROI and collecting data from the sensor nodes. In such frameworks, the consistent presence of a mobile sink in a ROI is not guaranteed. Utilizing a mobile sink helps to reduce the number of hops where the data are disseminated to the sink as it moves closer to the sender node. Although mobile sink-based solutions enable data gathering from remote places, in such applications, continuous monitoring is not possible without the help of a static infrastructure [34].

Aerial robots, such as unmanned aerial vehicles (UAVs), can play a significant role in mobile sink-based WSN data-gathering scenarios [35]. Unlike a ground-based mobile sink, an aerial mobile sink can fly along a guided route within a shorter time due to its three-dimensional movement capability [36]. These frameworks have the ability to perform without the help of any infrastructure, which enables data gathering from inaccessible terrain or even from sea surfaces [37]. Network congestion decreases as the aerial sink works as a data mule. Every time it collects and delivers data from the sensors to the base station (BS), the aerial mobile robot can be recharged. Considering these advantages, many researchers have proposed aerial robot-like, UAV-based data-collection optimization in WSNs [38,39]. Hybrid approaches have also been reported, which assume the presence of a static infrastructure in addition to a mobile sink. Instead of an aerial sink moving toward all the sensor nodes to collect data, one leader node is chosen by utilizing clustering or chain construction techniques. An aerial sink can fly directly to the WSN deployed zone and collect data from the cluster head (CH) or leader node [40].

Several review studies have been published on data-gathering techniques in WSNs. In [41], five energy-efficient data-gathering protocols for UAV-aided WSNs are discussed and compared qualitatively, mostly focusing on the main ideas and performance evaluation techniques. Focusing on the energy efficiency of WSNs, various data-collection approaches are discussed [42,43]. However, they did not consider mobile robot-based data collection. An overview of the mobile robot-based data-gathering process in WSNs and the corresponding issues and challenges are provided in [44]. The necessity of mobile robot trajectory optimization in the performance improvement of WSNs is discussed in [1] based on applications, trajectory techniques, and domains used to formulate the trajectory. Various data-gathering techniques in WSNs are presented, focusing on the optimization of important performance metrics such as network lifetime, latency, data accuracy, and energy consumption. A classification of mobile sink-based data dissemination strategies in WSNs along with their advantages and disadvantages is discussed in [45]. Considering data gathering based on a mobile sink, a survey on routing protocols in this area is presented in [46]. A range of techniques related to mobile robots in WSNs is reviewed in [47]. A qualitative review of fast and efficient mobile robot-based data-collection schemes in WSNs is provided in [48]. A survey on aerial mobile sink-based WSNs is presented in [49] in terms of different aspects of the integration of a mobile sink and a WSN. However, none of these surveys compare static- and mobile-sink-based data-gathering techniques. In addition, most of the survey works have been carried out based on qualitative performance analysis. Although a simulation-based analysis of the energy efficiency of WSNs with static and mobile sinks is described in [50], the aerial mobile robot-based sink is not explicitly considered. The main ideas of the exiting surveys on data gathering in WSNs are summarized in Table 1. However, there has been no quantitative performance analysis published to date, which has compared the performances of data-gathering schemes in WSNs considering the three types of sinks (i.e., static, ground mobile, and aerial mobile sinks). On the contrary, our paper reviews such data-gathering frameworks based on the three different types of sinks, analyzing the data-gathering frameworks both qualitatively and quantitatively.

**Figure 1:**
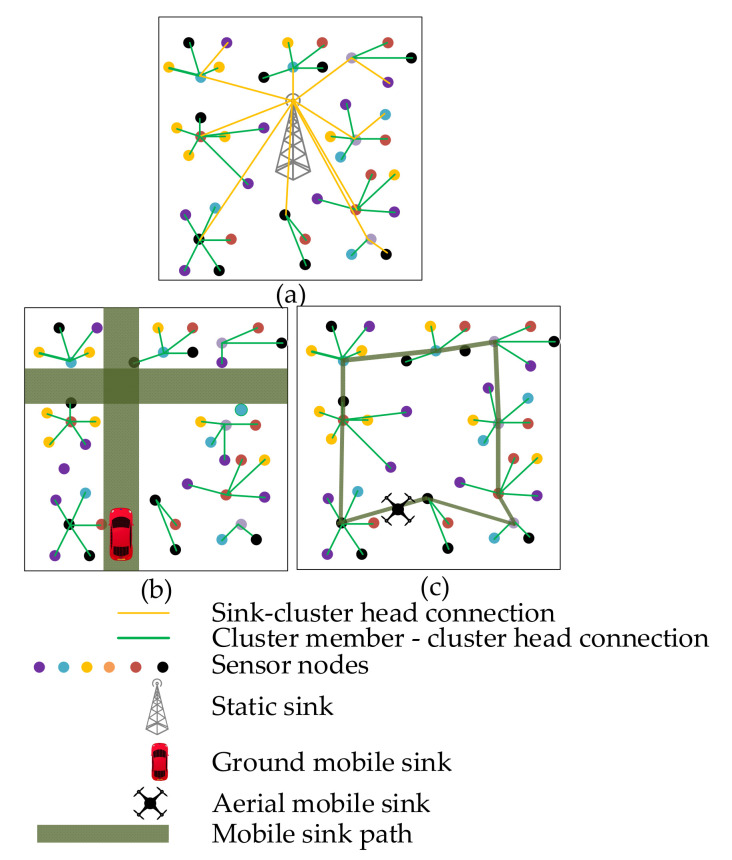
Data gathering in WSNs with (**a**) a static sink, (**b**) a ground mobile sink, (**c**) an aerial mobile sink.

The use of WSNs is increasing daily. However, collecting sensor data remains a major challenge. The lifetime of the WSN depends on the manner in which the perceived data are collected. Depending on the sink type, the lifetime of the sensor nodes and the quality of services (QoS) parameters of the network will vary. Therefore, suitable application scenarios and expected outcomes should be analyzed before choosing the sink type. Static sink-based solutions can be suitable options for long-term implementation. For a terrain where a ground robot can move, ground robot-based solutions can be applied. In some application areas, however, terrain may not be accessible to ground robots; thus, aerial robots come into play. Although the data-collection trajectory is easy to plan with the help of an aerial robot, they also have energy constraints and delay problems. The specific limitations of different types of sinks raise the importance of their comparative performance evaluation. This paper addresses this gap and presents comparisons that will reveal the relative performances among static sinks, ground robot-based mobile sinks, and aerial robot-based mobile sinks. Figure 1 shows the visualization of data gathering in WSNs based on different sink types.

The main contributions of this research are as follows:

- This paper presents both qualitative and quantitative comparisons among WSN data-gathering frameworks categorizing them based on their sink types (i.e., static sink, ground robot-based mobile sink, and aerial robot-based mobile sink). Instead of providing a comprehensive review of all the existing data-gathering frameworks for each category, this study selects four protocols for static sinks and three protocols for the other two categories to provide the fundamental understanding of each data-gathering paradigm.
- An overview of the data-gathering techniques is provided to afford a basic idea for each of the categories. The frameworks are individually described in terms of their working procedures, advantages, and disadvantages.
- A qualitative comparison among the data-gathering techniques of the three categories is provided by mentioning their main ideas, optimization criteria, and performance evaluation parameters.
- A simulation-based comparison was conducted to evaluate the performance of the data-gathering frameworks. One protocol from each category is selected for quantitative comparison in terms of energy efficiency, the number of dead nodes, control overhead, the number of packets dropped, and the delay.
- Finally, the lessons learned from the comparative analysis are presented. In addition, some recommendations for designing efficient WSN data-gathering frameworks are highlighted.

The remainder of this paper is organized as follows. In Section 2, the data-gathering frameworks are reviewed, along with their advantages and limitations. In Section 3, a qualitative comparison of the investigated protocols is provided in terms of their main ideas, optimization criteria, and performance evaluation techniques. In Section 4, the data-gathering frameworks are quantitatively compared via computer simulations in terms of the major performance metrics. In Section 5, the lessons learned from the study along with recommendations for proposing a robust data-gathering framework are presented under different application scenarios. We conclude this paper by summarizing our findings in Section 6.

## 2. Data-Gathering Frameworks Depending on Sink Types

In this section, the rationale behind choosing the protocols is discussed, and then the working methodology as well as the advantages and disadvantages of the different types of investigated sink-based WSN data-gathering frameworks are presented. The three categories are described in the following subsections.

### 2.1. Protocol Selection Criteria for Qualitative Comparison

This study examines three categories of WSN data-gathering techniques. First, total ten data-collection protocols are chosen for a qualitative comparison. As mentioned earlier, the protocols are chosen based on the sink types (i.e., static, ground mobile, and aerial mobile sinks). The protocols discussed in the static sink-based data-gathering section are chosen based on popularity and citations in the literature. To choose the ground and aerial mobile sink-based data-gathering techniques, we focus on journal articles. The mobility pattern of the ground mobile sink heavily depends on the terrain type. While choosing the protocols for ground mobile sinks and aerial mobile sinks, we also take the mobility pattern. Besides the aforementioned criteria, we also focus on maximizing the difference of the protocols for each section in terms of configuration and working principle.

### 2.2. Static Sink-Based Frameworks

Static sink-based solutions are the most popular WSN data-gathering solutions among the investigated categories. In these frameworks, a static sink is assumed to be either inside or outside the ROI, depending on the application scenario. The static sinks are assumed to have unlimited power sources and are connected to the control center.

#### 2.2.1. Threshold-Sensitive Energy-Efficient Sensor Network (TEEN) Protocol

Manjeshwar et al., proposed a threshold-sensitive energy-efficient data gathering and transmission protocol for reactive WSNs called TEEN [56]. In reactive networks, nodes respond immediately to sudden changes in the network environment attributes. In TEEN, data gathering is performed using a hierarchical clustering mechanism. Cluster formation is performed as in the low-energy adaptive clustering hierarchy (LEACH) protocol, which is a proactive network protocol. Unlike LEACH, TEEN is designed for reactive networks that are well suited for time-critical applications. The CHs of the uppermost levels of the hierarchy directly report to the BS. Energy consumption is reduced as the nodes only have to transmit to their immediate CHs, and the additional computation is only performed by the CHs. Moreover, CHs are rotated by every node working as a CH for a cluster period *T* in order to evenly distribute energy consumption. At each cluster period, the CHs broadcast a hard threshold ($H_{T}$) and a soft threshold ($S_{T}$) value to their members along with the attributes. The sensor nodes consistently sense the environment. When a parameter from the attribute set is equal to its $H_{T}$ value, the sensed data are stored in a variable called the sensed value (SV). When the current value is higher than $H_{T}$ and differs from the SV by more than the $S_{T}$ value, the node transmits data. Subsequently, the SV is changed to the current value of the sensed attribute. The attributes are set to new values during broadcasting in every clustering period. The value of $S_{T}$ can be adjusted based on the requirement, with a trade-off between the accuracy of the data and energy consumption of the network.

Advantages: The CH rotation after each cluster period balances the energy consumption of the network to maintain the overall network lifetime. Using the $H_{T}$ and $S_{T}$ values leads to a decrease in the number of data transmissions by the sensor nodes.

Limitations: As the nodes transmit data only when the $H_{T}$ and $S_{T}$ conditions are met, the transmission may never occur if one of the conditions is not checked. Although the rotation of the CH balances the energy consumption in the network, repeated clustering at each interval may not be computationally efficient.

#### 2.2.2. Power-Efficient Data Gathering in Sensor Information System (PEGASIS)

**Figure 2:**
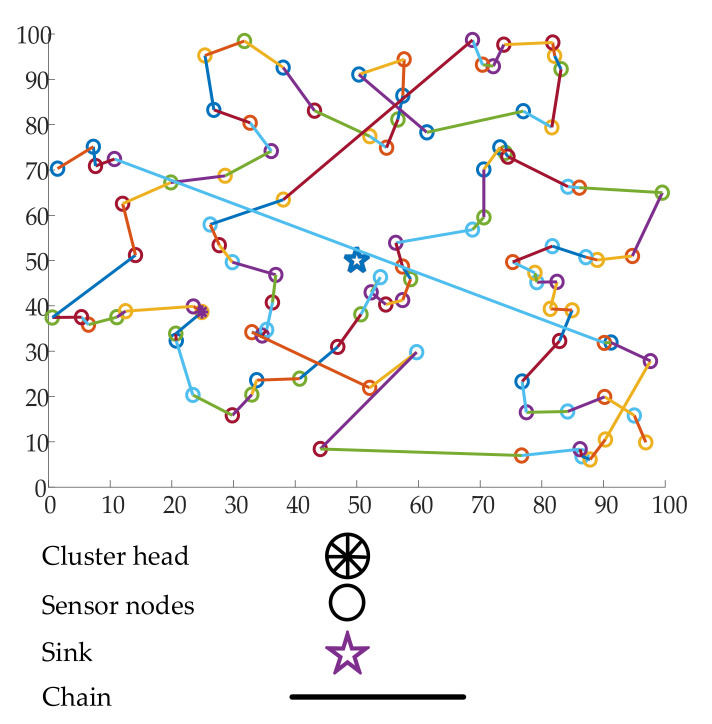
Working mechanism of the PEGASIS protocol.

Lindsey et al., proposed PEGASIS [57], which is an energy-efficient data-gathering mechanism for static sink-based WSNs. PEGASIS is an extension of the well-known LEACH clustering algorithm. In LEACH, the aggregated data in the CH are sent to the sink in a direction transmission manner. PEGASIS adopted a multi-hop approach to deliver data to the sink. The primary purpose is to reduce the transmission distance for every sensor node, resulting in energy-efficient data transmission. The node deployment is assumed to be random in this case. The chain formation among the sensors is performed either by the sensors themselves or by the sink node. In the case of chain formation by the sink nodes, the sink node computes the chain information and broadcasts it to the node. The primary assumption of this algorithm is that the sink node is far away from the ROI. The chain formation algorithm is a greedy algorithm. Another major assumption of PEGASIS is that the data fusion capability belongs to every node in the network. When a node receives data from any other node, it fuses the data with its own and then transmits the data to the next node. The node that transmits the data to the sink is chosen randomly from the WSN. This is done to ensure that the nodes are dying from different positions of the chain. Figure 2 shows the working mechanism of the PEGASIS protocol. As shown in the figure, the original PEGASIS algorithm forms one chain and selects one leader/CH node for each round of transmission.

Advantages: The mechanism adopted in PEGASIS is heavily dependent on the aggregation percentage. However, assuming that the nodes have low homogeneity, PEGASIS will form a load-balancing mechanism in terms of energy consumption.

Limitations: Despite having a better load-balancing mechanism, the leader node in a chain still needs a higher transmission power to deliver the data to the sink. The assumption about global knowledge is costly in terms of memory and beaconing. The strict one-packet forwarding mechanism assumption is not realistic; the nodes may need more data packets to transmit the fused data.

#### 2.2.3. Hybrid Energy-Efficient Distributed (HEED) Clustering

**Figure 3:**
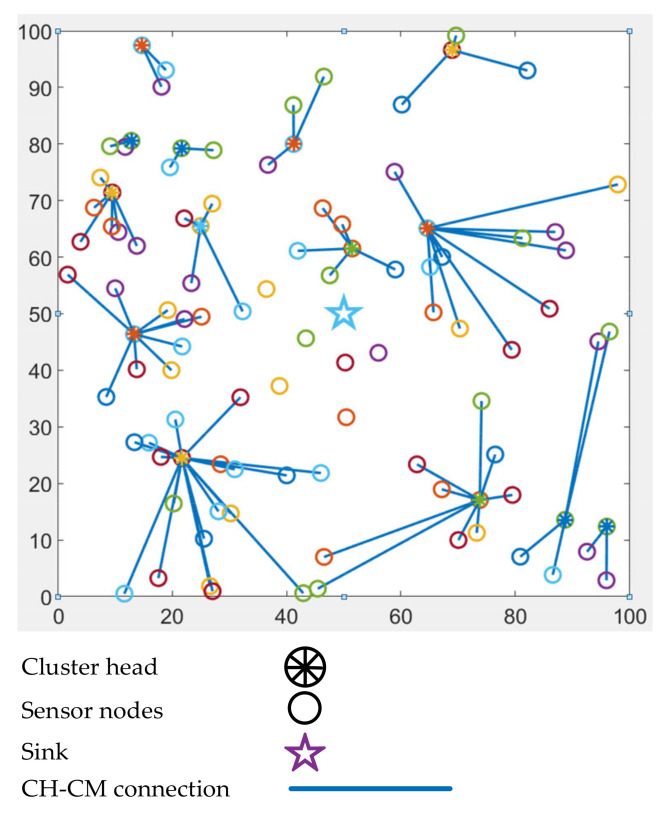
Working mechanism of the HEED clustering algorithm.

Younis et al., proposed a distributed clustering algorithm called HEED [58]. This algorithm works in a distributed manner inside every sensor node and forms a cluster with a CH. HEED is a clustering algorithm that has been proposed for homogeneous sensors. The primary assumption of this clustering algorithm is that the sensor nodes have multiple transmission mechanisms. To model the algorithm, the authors used the parameters from Berkeley Motes [59]. The HEED algorithm operates in three phases: initialization, main processing, and finalization. In the initialization phase, the algorithm forms a neighboring set, computes its own cost, and broadcasts it to its neighbors. The CH probability is also assigned in this phase. The following equation is used to compute the probability of the CH:

(1) $$CH_{p}=\max\left(C_{p}\times \frac{E_{res}}{E_{M}},p_{min}\right)$$

where $CH_{p}$ is the CH probability, and $C_{p}$ is a random number. The number of CHs depend on the value of $C_{p}$. $p_{min}$ is the minimum value assigned to nodes when the value of the residual energy goes beyond a threshold limit. $E_{res}$ is the residual energy, and $E_{M}$ is the maximum amount of energy that a node has after deployment. The primary parameter for CH selection is the energy, the secondary parameter being the node degree in the case of the fixed transmission power inside a cluster. Based on the node degree, HEED has the ability to balance the number of cluster members (CMs) in a cluster. When the power range is a variable for intra-cluster communication, the cost is determined by the minimum average power of all the nodes of a CH. In the iteration phase, HEED attempts to determine the final CH from the list of candidate CHs a node has within its vicinity. If a node does not find any tentative CH in its transmission range, it simply declares itself as the CH. The probability of the CH doubles in every iteration. When the value of $CH_{p}$ reaches one, a tentative CH declares itself as the final CH. The sensor nodes ultimately select a CH and join the cluster. Finally, if a sensor node does not find any final CH, it declares itself as the final CH and completes the clustering process. Figure 3 shows the working mechanism of the HEED clustering algorithm. The figure shows the basic clustering outcome using the HEED algorithm. It should be noted that some nodes are not left alone and do not join any cluster. This is shown in order to depict the trivial single CH cluster problem of the HEED algorithm.

Advantages: This protocol does not utilize assistance from any positioning system. It selects neighbors based on the received signal strength indicator (RSSI) value. As a distributed algorithm, the deployment of sensor nodes is possible in remote areas.

Limitations: The proposed algorithm increases the chance of developing a single-node CH. A single-node CH directly transmits the sensed data to the sink. This requires a long-range transmission. In addition, a sensor node could have changed its transmission power level and may join another CH to remove rapid energy depletion.

#### 2.2.4. Collection Tree Protocol (CTP)

Fonseca et al., proposed a tree-based routing protocol called collection tree protocol (CTP) [60]. In CTP, the tree roots are determined after the advertisements of some nodes, and a set of routing trees are created using these roots. A parameter named expected transmission (ETX) value based on the distance from the root is used to calculate the cost of routing. A routing loop problem occurs when a node receives the data packet from another node that has an equal or lower ETX value. Two mechanisms are used to solve this problem. First, upon detecting the inconsistency in the ETX value, CTP broadcasts a beacon frame so that the sender node can receive it and adjust its routes. Second, CTP does not select a route that has an ETX value higher than a predetermined constant value. Another problem of CTP is packet duplication which occurs due to the retransmission of the same packet. To resolve this problem, CTP monitors a packet for duplication before forwarding, upon receiving the packet. A cache of packets is utilized to detect the duplication and the packet is discarded if it is a duplicate. However, due to the routing loop, a node may receive a packet more than once, which creates complications for packet dropping because of duplication. Therefore, a parameter called ‘time has lived’ (THL) is contained in data frames, which is incremented in each routing loop. The CTP data frame contains the ETX, THL, origin id of the packet, origin sequence number, data payload, higher-level protocol identifier, and two control bits named pull bit and congestion bit. In the CTP routing frame along with the ETX, pull bit, and congestion bit, the node’s current parent is provided. For implementing CTP, a link estimator, a routing engine, and a forwarding engine are required. The routing engine calculates the ETX for communicating with single-hop neighbors. The routing engine selects the next-hop neighbor with the help of link estimation and network-level information. The forwarding engine takes the decision of packet sending from the queue of packets.

Advantages: The main advantage of CTP is that it can send data to the sink nodes using the route with the minimum cost, in which the cost depends on the number of hops. Therefore, the total of hops can be reduced in CTP, which makes a routing protocol efficient. Moreover, it can adapt to dynamic topology changes by adjusting the routes.

Limitations: CTP is suitable for comparatively low traffic rates and, thus, it may not achieve good performance in a network of high traffic rates. Hence, it is suitable for a WSN environment with a large number of nodes sending packets simultaneously.

### 2.3. Ground Mobile Sink-Based Frameworks

In this category, the frameworks assume a single or multiple ground robot that acts as a mobile sink. The robot can follow a trajectory, moving closer to the transmitting nodes, which results in a shorter transmission range and less energy consumption as data transmission is reduced. However, the delay is likely to increase. The terrain should be accessible for the robot to move freely.

#### 2.3.1. Circular-Pathway Mobile Sink-Based WSN Data Collection (CPDC)

Wang et al., proposed a mobile sink-based WSN data-collection mechanism [61], in which the geographical area is divided into different sectors. Depending on the fitness value, each sector contains a CH whose primary responsibility is to perform the data-collection task inside its corresponding sector and relay the data to the mobile sink after the primary calculation. The sectors have the same geographical area. The fitness of being a CH depends on the distance and energy level of a node and its distance to the data sending nodes. A node willing to send data to the CH can decide to send data via a multi-hop or single-hop data transmission, depending on which is the most optimal method.

**Figure 4:**
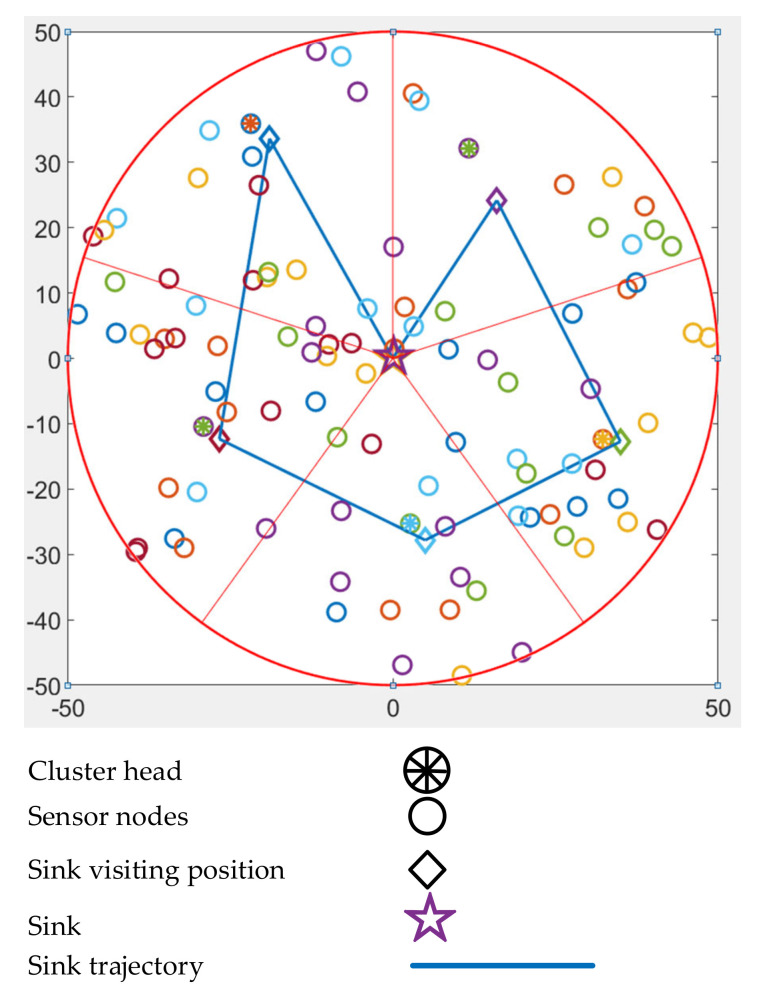
Working mechanism of the CPDC protocol.

In terms of the energy-consumption policy, the CHs are connected in a greedy manner. The CH node that is nearest to the sink node is selected as the gateway node to disseminate the data. This mechanism follows a predefined trajectory for a mobile agent. The sensor nodes are equipped with a GPS mechanism. Each sector has a fixed angle value, which can be derived as follows:

(2) $$A=\frac{2\pi }{N_{CHs}}$$

where the number of CHs is expressed by $N_{CHs}$, and A denotes measurement of the angle. The main reason for using the mobile sink is to reduce long-distance communication, which consumes a significant amount of energy. To establish communication among the clusters, a greedy policy is applied to conserve the energy of the WSN node. At the beginning of the process, the sink node queries all the CHs in the network. After collecting all the replies, it elects the gateway node. All the CHs are willing to transmit data sent to the CHs designated by the sink, which are geographically closer to the gateway node. The robot maintains a fixed speed during the data-collection mission. Figure 4 shows the working mechanism of the CPDC protocol.

Advantages: The sink maintains a constant velocity. This mechanism enables the nodes to calculate the position of the sink after a certain amount of time. As a result, the sink does not need to broadcast its position repeatedly to the sensor nodes.

Limitations: The CHs are connected in a greedy manner with each other, but no solution is provided for an isolated cluster member. This mechanism assumes that every node has location information about other nodes, which is not a good assumption, as the sensor nodes are inexpensive and do not have a large buffer memory. For a large and dense deployment, this information will be an extra burden on the sensor node’s memory.

#### 2.3.2. Integration of Geographic and Hierarchical Routing

**Figure 5:**
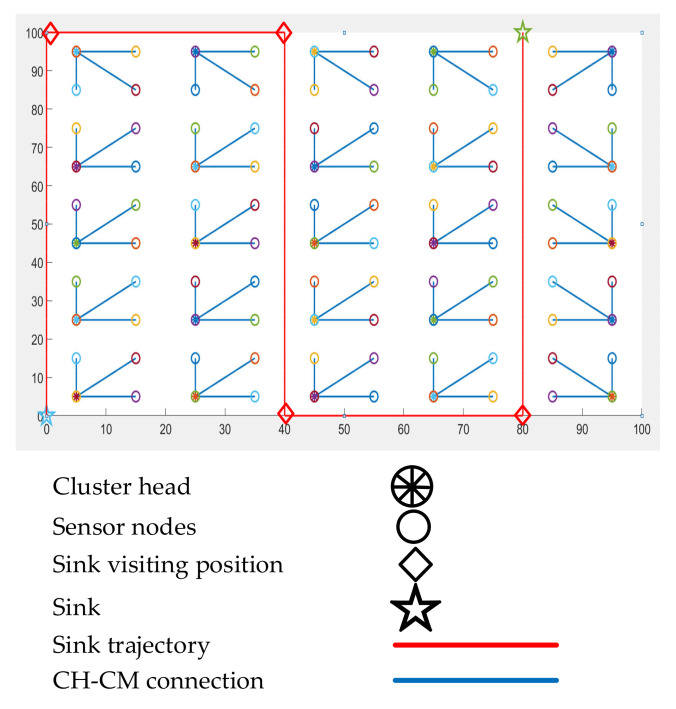
Working mechanism of the IoJHR protocol.

Vahabi et al. [62] proposed a hierarchical and geographic-based WSN data-collection mechanism IoJHR, with the help of a mobile sink. The main objective of this research was to establish energy-efficient data collection, resulting in an increased WSN lifetime. The network model used in this approach utilizes a virtual grid approach. Each virtual grid consists of a CH and four other sensor nodes. The CH is responsible for disseminating the data to the sink node. The energy level is used to elect the CH from a virtual grid. The number of virtual grids could be determined as follows:

(3) $$N_{virtualgrids}=\frac{N}{4}$$

where the number of virtual grids is denoted by $N_{virtualgrids}$, and *N* represents the number of sensor nodes. At the beginning of the data-collection operation, the sink node broadcasts its position in the ROI. During the rest of the operation, all the CHs know the exact position of the sink node as the trajectory of the sink is predefined. In fact, the sink node does not need to send its positional information twice. Figure 5 shows the working mechanism of the IoJHR protocol.

The control system operates a data-collection mission based on fixed-time scheduling. As all the sensor nodes are aware of the scheduling, the nodes can easily predict the next arrival time of the sink node. To collect data, the mobile sink moves back and forth, based on predefined time scheduling. When a CH finds the sink closer to its position, it simply transfers its data to the sink. This mechanism supports data collection via multiple sinks. However, to enable a new sink in action, the predefined path and collection time should be conveyed to the sensors beforehand. In the case of multiple sink scenarios, CHs are divided into different sinks for data offloading.

Advantages: This procedure applies techniques to support multiple sink nodes. As a result, the latency for data collection decreases dramatically.

Limitations: It is assumed that every virtual head (VH) has exactly four nodes. This assumption is not practical for random or unplanned deployment. Even in the case of planned deployment, depending on the spatial necessity for sensing, four nodes may not be in close proximity to each other. In such a situation, if a cluster is about to form consisting of four sensor nodes, energy consumption will increase due to long-distance data transmission. There are no inter-cluster communication mechanisms mentioned in this study.

#### 2.3.3. Energy-Efficient PSO-Based Routing with Mobile Sink (EPMS)

Wang et al., proposed EPMS, an energy-efficient clustering scheme for WSNs with mobile sinks [63]. They used a particle swarm optimization (PSO) algorithm for clustering. PSO is an optimization method inspired by swarm intelligence that falls under the category of artificial intelligence. Initially, the sensor nodes forward their position and energy information to the BS. Using this information, the PSO algorithm divides the entire network into clusters and defines the K particles. By creating a region partition line, the entire network is divided into 2K K sub-regions. The fitness value *F* for K particles can be computed as follows:

(4) $$F=\alpha \sqrt{\overset{2}{\underset{i=1}{\sum }}{\left(c_{i}-f_{i}\right)}^{2\;}}+\beta \sqrt{\overset{2}{\underset{i=1}{\sum }}{\left(\frac{E_{i}}{c_{i}}-\frac{E_{sum}}{N}\right)}^{2\;}}$$

where F denotes the fitness value, $c_{i}$ is the number of sensor nodes in region *i*, N is the total number of sensor nodes, $f_{i}$ is the number of CHs in each cluster, $E_{i}$ is the total energy consumption in region *i*, and $E_{sum}$ is the total energy. Here α and β are the weight factor and the relation is, (α+β) = 1. The fitness values are evaluated, and the parameters of the particles are updated accordingly. The algorithm iterates until F converges to the minimum values, and *M* clusters are created. Subsequently, CH selection is performed for each cluster based on the residual energy of the sensor nodes. The node with the maximum residual energy in a cluster is selected as the CH. The selected CH is maintained until the next round of energy saving in the network. The data-collection round is completed when the mobile sink visits all the CHs in the network.

Three types of data packets are used in the EPMS. First, the Hello packet—which consists of a cluster ID number, average residual energy, CH location, and time period—is used to determine the CH that sends data to the mobile sink. The second packet, Message-c, contains data for directly transmitting to the sink node. Finally, the Message-m packet is used to send information to the CH. The CH sends the collected data to the mobile sink when it moves to the CH. This process is repeated for all the CHs in the network.

Advantages: The CH selection is carried to the next data transmission round, which helps to balance the network energy consumption. Unlike other studies, the packet delivery ratio and latency are evaluated for EPMS.

Limitations: Clustering and CH selection are performed solely based on the residual energy of the sensor nodes. The transmission distance is not considered for CH selection, but it can have a negative impact on data gathering from the CH.

### 2.4. Aerial Mobile Sink-Based Frameworks

This category is the newest among the investigated sink types. UAVs are being used and tested for different application scenarios, one of which is the collection of WSN data. To collect WSN data, both multirotor and fixed-wing UAVs have been proposed in the literature. Multirotor drones have the ability to fine-tune data-gathering positions, and these drones can hover in a steady position. However, compared to multirotor drones, fixed-wing UAVs are faster and consume less energy. Therefore, for precise data collection, multirotor UAVs are used, and for fast data collection, fixed-wing UAVs may be employed.

#### 2.4.1. Node Density-Based Clustering and Mobile Collection (NDCMC)

Zhang et al., proposed a mobile sink-based data-collection mechanism called NDCMC for WSNs [64]. NDMC employs a CH selection mechanism and a trajectory optimization algorithm for the mobile sink. This algorithm also aims to increase the lifetime of the WSN by reducing the transmission energy and by planning an optimum data-collection path for the mobile agent. NDCMC assumes the presence of GPS information for both the mobile agent and sensor nodes. In addition, this protocol assumes the presence of a static infrastructure, and that the sensors are also provided with information prior to deployment. To inform the presence of the mobile agent, a periodic beacon message is broadcast.

**Figure 6:**
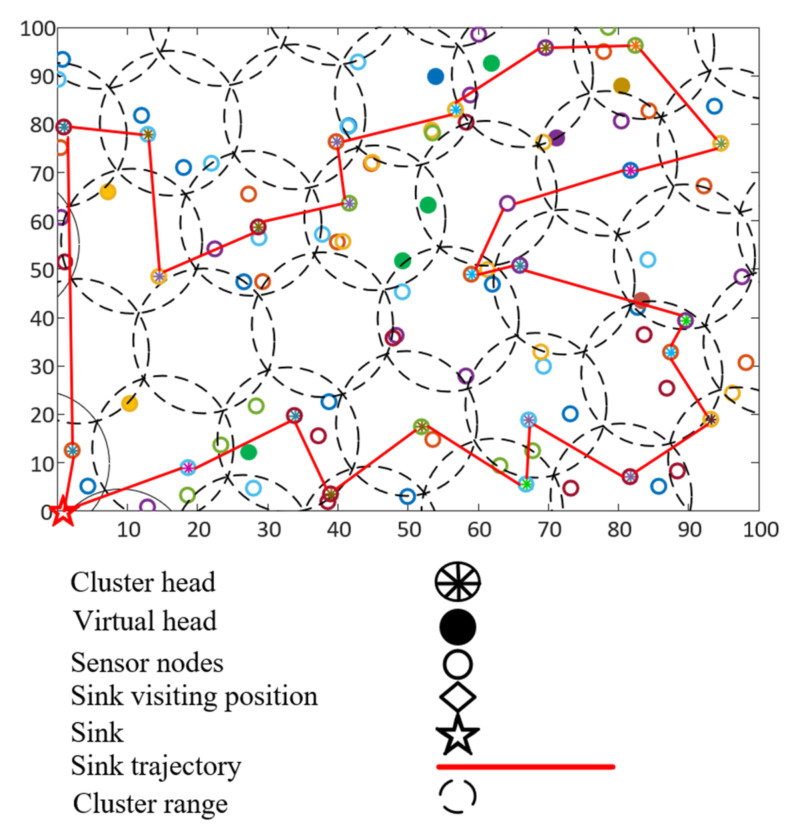
Working mechanism of the NDCMC protocol.

Initially, all nodes broadcast “hello” messages by attaching their ID and positional information. In this way, all nodes obtain information about other deployed nodes. With the help of a geographic routing mechanism, all sensor nodes deliver their own information to the BS in a multi-hop fashion. Based on the received information, the BS elects the CHs from the densest zone and plans the tour of the mobile agent accordingly. NDCMC introduces another type of node, called a VH. Nodes that are inside the communication range of the mobile agent but are not CHs are called VHs. After electing the CHs and VHs, the mobile agent informs the entire WSN about a specific node’s role. Upon receiving the information, the normal nodes locate their closest CH or VH. Figure 6 shows the working mechanism of the NDCMC protocol.

Both CHs and VHs are responsible for gathering data. However, the trajectory of the UAV is only calculated based on the CHs, and this is the only difference between the VHs and CHs in NDCMC. In the NDCMC process, the CHs are mostly located at the center of a circular area. When the energy level of a CH falls below a threshold level, the CH becomes an ordinary node and broadcasts the message to the local nodes in its vicinity. Nevertheless, the mobile agent still enters the ROI, and the ordinary nodes transmit their data in a direct hop fashion. After the death of a CH, its one-hop member starts to play the role of a VH. The expected number of VHs can be expressed as follows:

(5) $$N_{v}=4Rr\left(\frac{N_{s}}{A}\right)-1$$

where $N_{s}$ is the number of sensor node, $N_{v}$ is the expected number of VHs, the length of the mobile agent’s path is 2*R*, the VH and mobile agents’ distance is *r*, and *A* is the area covered by the mobile agent.

Advantages: The trajectory of a mobile sink is not static and depends on the density of the node. This mechanism helps the majority of the nodes reduce their transmission energy cost. Isolated nodes are declared as CHs by the BS, and the mobile agent is given extra responsibility for visiting the node.

Limitations: Initial “hello” messaging increases the number of exchanged control packets. As the assumption of NDCMC is that of the dense deployment of the WSN nodes, the initial “hello” message will also have an adverse impact on the buffer memory of sensor nodes.

#### 2.4.2. UAV-Enabled Energy-Efficient WSN Data Collection (UEWDC)

Zhan et al. [65] proposed an energy-efficient UAV-based WSN data collection technique named UEWDC. A joint optimization problem was studied with the UAV’s trajectory and the wake-up scheduling of sensor nodes. The mixed-integer non-convex optimization problem was relaxed and solved by obtaining a suboptimal solution. The main goal was to minimize the maximum energy consumption of the overall sensor nodes while simultaneously maintaining the target volume of reliable data collection. The authors considered a general fading channel model for the link between the sensor node and the UAV. It is assumed that the initial location $q_{o}$ and ending location $q_{F}$ of the UAV’s trajectory are predetermined, and there exists at least one feasible flying path from $q_{o}$ to $q_{F}$. Moreover, the sleep and wake-up mechanism of sensor nodes is designed such that a maximum of one sensor node can be in wake-up mode at each time slot for communicating with the UAV. The nodes remain in sleep mode most of the time and wake up only when the UAV is sufficiently close. The relaxed problem with successive convex optimization is solved using an iterative algorithm to determine the suboptimal solution of the UAV trajectory and wake-up schedule.

Advantages: This work is suitable for sparse network conditions, and when the position of nodes is known. The energy consumption is balanced by maintaining the sensor nodes in sleep mode. For a WSN with periodic data gathering, this scheme balances energy consumption and reliable data delivery.

Limitations: The proposed method is not compared with any other optimized method. The number of nodes considered for the system is very low, and the manner in which the trajectory and wake-up schedule work in a dense network has not been discussed. This mechanism is not suitable for environments with emergency data collection.

#### 2.4.3. UAV-Based Compressive Data Gathering (UAVCDG)

Ebrahimi et al. [66] proposed an energy-efficient UAV-based WSN data-gathering mechanism named UAVCDG. First, a joint optimization problem of sensor node clustering, data forwarding tree construction, CH selection, and UAV trajectory planning is formulated for data gathering in dense WSNs. The joint optimization problem can be written as follows:

(6) $$Minimize\;\omega \underset{\begin{array}{c} m\;\epsilon \;W\\ h\;\epsilon \;V\\ \left(i,j\right)\epsilon \;E \end{array}}{\sum }P_{ij}^{m,h}+\left(1-\omega \right)\;\underset{i\;\&\;j\;\epsilon \;\left\{V\cup S\cup D\right\}}{\sum }d_{ij}U_{ij}$$

The first part of the problem refers to minimizing the total transmission power of the active links in the forwarding trees, where $P_{ij}^{m,h}$ is the transmission power required to transmit data from node *i* to *j* in cluster *m* when *h* is the CH. *V* is the set of sensor nodes, *E* is the link between nodes, and W is the set of clusters. The second part refers to the trajectory distance of the UAV where $U_{ij}\;\epsilon \;\left\{0,1\right\}\;$ indicates whether the UAV will fly from node *i* to *j*, and $d_{ij}$ is the distance between nodes *i* and *j*. *S* is the starting point, and *D* is the destination sink node. ω is a weight parameter for each part that can be adapted. The constraints regarding to the optimization problem can be found in [66]. The joint problem is decomposed into four complementary subproblems to achieve near-optimal results with low complexity. The network environment is considered to be a rural area with a line-of-sight link, which means that no obstacles exist in the network. A clustering technique is applied among the sensor nodes to shorten the trajectory of the UAV and balance the energy consumption. The aim of clustering and CH selection is to jointly minimize the overall transmission power for data gathering and UAV trajectory. A compressive data-gathering (CDG) technique is utilized to minimize the amount of data transmission to optimize energy consumption. Through CDG, an encoded sum of the gathered data is received by the sink node, which can be decoded to the original data. To solve the first subproblem of clustering, a K-means clustering algorithm is implemented, and the clusters are updated to almost equal-sized cells. Subsequently, an algorithm is established for CH selection that uses the starting position *S* and destination position *D* of the UAV’s trajectory to select the CH with the shortest distance from the path. To solve the UAV trajectory subproblem, a heuristic algorithm—called the nearest-neighbor algorithm—is proposed. Finally, to solve the subproblem of forwarding tree construction in each cluster, a minimum spanning tree method is considered.

Advantages: The CDG technique distributes the transmission load across several sensor nodes in the network. This distribution balances energy consumption with an extended network lifetime. The performance evaluation for small, medium, and large network scenarios demonstrates the efficiency of the proposed method.

Limitations: The residual energy of the sensor nodes is not considered, which is especially important for choosing the CH. Only UAV trajectory minimization is considered for clustering and choosing the CH. Selecting a CH with low residual energy may cause the death of the node, leading to failure in data gathering.

## 3. Qualitative Comparison

This section presents a comparative study of the reviewed frameworks by discussing the main ideas, optimization criteria, evaluation parameters, and performance evaluation techniques.

### 3.1. Main Idea of the Frameworks

Each of the data-gathering frameworks has unique properties that are the main performance-controlling features of that scheme. The main ideas of the techniques reviewed in this study are compared in Table 2.

### 3.2. Optimization Criteria and Evaluation Parameters

The investigated schemes are intended to optimize the data-gathering process in WSNs from different perspectives. Table 3 shows a comparison of the optimization techniques utilized, and the performance evaluation parameters considered in the reviewed schemes. An implementation of CTP with performance evaluation has been provided in [67]. However, to be efficient for WSNs, the most crucial feature of a data-gathering protocol is minimizing energy consumption and thereby extending the network lifetime [68]. The sensor nodes are mostly powered by a non-rechargeable battery. Energy is consumed via sensing, internal calculations such as aggregation, and signal transmission. Consequently, different schemes have been attempted to minimize energy consumption in different ways, as shown in Table 3. TEEN and PEGASIS techniques consider the rotation of the CH and leader node, respectively, which means that all sensor nodes participating in becoming the CH or leader node, distributing energy consumption evenly in the network. Along with the communication cost from the CH to the sink node, the HEED protocol also considers the intra-cluster communication cost, which is responsible for the energy consumption in the network. The CPDC protocol limits re-clustering in the network under the condition of maximal weight. With the IoJHR protocol, data gathering from sensor nodes to the mobile sink is performed by only two hops in each virtual grid. One hop is for sensor nodes to the CH and another for the CH to the mobile sink, reducing transmission energy consumption. For energy balancing in the EPMS protocol, the selection of a CH is carried on to the next round of data transmission. NDCMC uses a VH along with a CH—which is used when a CH is out of power—rather than selecting another CH. UEWDC minimizes energy consumption by minimizing the node wake-up schedule, in which only one node wakes up at each transmission time. The goal of UAVCDG is to shorten the UAV trajectory to minimize the communication energy of the UAV.

The data-gathering techniques in the reviewed schemes are performed mostly by different clustering techniques, except for the PEGASIS, UEWDC, and CTP protocols. Data gathering through clustering helps to reduce the number of transmissions in the network. The data are aggregated in the CH and then transmitted to the sink node—instead of data transmission to the sink node via all of the sensor nodes—which reduces the overall network energy consumption. PEGASIS performs data gathering by chain formation among the sensor nodes via data fusion from neighboring nodes. UEWDC conducts energy-efficient data gathering by proposing a suboptimal solution for UAV trajectory minimization and node wake-up scheduling. None of these investigated protocols considered sensor node mobility, although the mobility of sensor nodes can be an issue in WSNs. Location awareness means that the location information of CHs or sensor nodes is required for data gathering to the sink node. However, the locations of sensor nodes are required only in protocols where sink nodes have mobility. The mobile sink node must traverse the sensor nodes to collect data. The energy consumption in the sink node is also an issue, especially in the case of mobile sink nodes, which are battery-powered, similar to the sensor nodes. Therefore, energy constraints in the sink node must be considered, but only UAVCDG considers sink node energy constraints.

The residual energy is the remaining energy of a sensor node. While selecting a node for data transmission, the residual energy consideration is necessary, as it has a significant impact on the network lifetime. The HEED, CPDC, IoJHR, EPMS, and NDCMC protocols consider the residual energy of a sensor node for CH selection. Selecting the node with the highest residual energy as the CH of a cluster helps in transmitting data through that CH for a longer time. The number of hops while optimizing data gathering in WSNs also increases the network lifetime significantly [69]. However, IoJHR and CTP consider the minimization of the number of hops in their data-gathering process. Clustering minimizes the number of hops compared to the traditional gathering of data from sensor nodes to sink nodes when the source node is far removed from the sink node.

Consideration of the proximity of a neighbor node in the design of a data-gathering technique helps to reduce energy consumption for data transmission from one sensor node to another. PEGASIS, HEED, CPDC, and NDCMC protocols consider the neighboring node proximity while performing clustering or CH selection in their data-gathering schemes, whereas CTP considers the proximity of the neighbor nodes for forwarding the data packet. A node is assumed to be dead when it loses power and cannot participate in sensing or data transmission. The transmission distance mentioned in Table 3 refers to the distance required for the transmission of data from the source node to the sink. The energy consumption increases with increasing distance, particularly in the case of a mobile sink when the sink has to travel to the sensor nodes for data gathering. All the reviewed protocols, except for the TEEN and EPMS protocols, consider the data transmission distance for their data-gathering schemes.

The investigated data-gathering protocols evaluate their performance by considering different parameters, of which energy efficiency is the most important evaluation parameter as energy consumption remains one of the most critical problems. All of the investigated protocols—evaluated by means of energy consumption—demonstrated that their methods exhibited better energy efficiency than the protocols to which they were compared. Consideration and evaluation of network lifetime are necessary when designing a data-gathering protocol—the network lifetime can be calculated using the data transmission duration or round until all nodes are alive or the first sensor node in the network dies. Apart from CTP and UEWDC, all the investigated protocols consider network lifetime, and the validation is done by comparing with other existing protocols. Network lifetime using different numbers of nodes was also analyzed.

In a WSN environment, where critical and emergency data gathering is required, latency is an important issue that needs to be taken into account. The CTP considers reducing the topology repair latency when the route is repaired after detecting an inconsistency in the network. This latency plays a role for reducing the overall data-gathering latency in the network. The IoJHR protocol evaluates the data-gathering latency with respect to a different number of nodes, comparing it with other protocols. The EPMS protocol has shown that the average data delivery latency decreases when the speed of the mobile sink increases. NDCMC performs a trade-off between power saving and data-gathering latency by adjusting the cluster radius. The packet delivery ratio is the total number of packets sent until the end of a simulation round. The IoJHR protocol evaluated the delivery ratio with 100, 200, and 300 nodes in the network and compared it with other protocols. The EPMS protocol compared its packet delivery ratio over an increasing number of rounds and compared it with other data-gathering schemes.

The IoJHR protocol evaluated the number of dead nodes in a network with respect to a different number of nodes. It was shown that the IoJHR protocol had a minimum number of dead nodes compared to other protocols. The NDCMC protocol defines a node as a dead node when its residual energy is less than a threshold. Subsequently, the number of dead nodes was evaluated by increasing the number of simulation rounds, the number of simulation rounds being a parameter that defines the network lifetime. The PEGASIS protocol appraised the number of rounds until 1%, 20%, 50%, and 100% of the nodes in the network were dead. The HEED protocol evaluated the number of rounds until the first node died, the last node lying with a different number of nodes in the network, after which the performance was compared with other protocols. The TEEN, CPDC, EPMS, and NDCMC protocols evaluated the number of nodes alive in the network with respect to the data-gathering round. Over time, the residual energy of the sensor node decreases.

## 4. Quantitative Performance Comparison via Simulation

To evaluate the performance of the investigated WSN data-collection methods, we used MATLAB simulations. The parameters used in the simulations are listed in Table 4. In this section, we first discuss the protocol selection criteria, energy model, and delay mode. We then critically analyze the simulation performance outcomes.

### 4.1. Protocol Selection Criteria for Quantitative Comparison

Three protocols are chosen for simulation from each category discussed above. The protocols are chosen such that they would maximize the differences between the core techniques and also represent the concepts of their specific categories; as a result, the simulation values represent meaningful information. From the static-sink category, we choose the HEED protocol for evaluation. This protocol is a distributed static sink-based framework that does not utilize any assistance from GPS. The clustering technique is performed solely by the sensor nodes themselves, and no assistance is provided by the BS. Conversely, we choose the IoJHR protocol from the ground robot-based sink category, a protocol which utilizes GPS. The sink node follows an S-path mobility model, and the planned deployment of the sensor nodes is assumed. The S-path is a static mobility model, and as a ground robot-based solution, the architecture does not exhibit ease of design for mobility based on specific parameters. As the ground structure may vary based on the terrain, it is often impossible for a ground robot to access hard-to-reach areas. Finally, the NDCMC framework designs sink node mobility based on the CH position. However, the CH is selected by the BS.

### 4.2. Energy Model

We adopted the energy model discussed in [70]. Energy depletion through data transmission can be derived using the following equation:

(7) $$E_{T}\left(l,\Delta \right)=E_{T-el\left(l\right)}+E_{T-amp\left(l,\Delta \right)}\;=\left\{\begin{array}{c} l*E_{el}+l*\varphi_{fp}*\Delta^{2},\;\;\;\;\;\;\;\Delta <\Delta_{th}\\ l*E_{elec}+l*\varphi_{mw}*\Delta^{4},\;\;\;\;\;\;\Delta >\Delta_{th} \end{array}\right.,$$

where the energy consumption due to signal transmission is denoted by $E_{T}$. l denotes the length of the packet, and Δ represents the distance between the transmitter and receiver. Energy depletion due to circuitry operation for a data packet containing l bits transmission is given by $E_{T-el\left(l\right)}$. The transmitter energy consumption model due to free-space propagation is denoted by $\varphi_{fp}$, and for multipath propagation, the model is denoted by $\varphi_{mw}$. $\Delta_{th}$ is the threshold distance for determining free-space propagation and multipath propagation. The threshold distance can be calculated as follows:

(8) $$\Delta_{th}=\sqrt{\frac{\varphi_{fp}}{\varphi_{mw}}}$$

Energy depletion for receiving l bit of energy can be expressed as follows:

(9) $$E_{R}\left(l\right)=E_{R-el}*l,$$

where $E_{R}\left(l\right)$ is the energy consumption due to receiving l bits of data. $E_{R-el}$ represents the energy consumption due to receiving one-bit data.

### 4.3. Delay Model

To analyze the delay, we only consider the sink mobility delay of the proposed algorithm. The data propagation delay is less significant compared to the sink mobility delay. To compute the delay, we consider the model expressed by the following equation:

(10) $$\overset{R_{n}}{\underset{r_{n}=1}{\sum }}\left(\frac{1}{V_{du}}\left(\Delta_{\left(S,P_{1}\right)}+\Delta_{\left(P_{\left|P\right|},S\right)}+\overset{\left|P\right|}{\underset{i=1}{\sum }}\Delta_{P_{i},P_{i+1}}\right)\right),$$

where $V_{du}$ is the default velocity of the mobile sink, $\Delta_{\left(S,P_{1}\right)}$ is the distance between the first position $P_{1}$ and the starting position in the ROI (S), $\Delta_{\left(P_{\left|P\right|},S\right)}$ is the distance between the final position of the UAV, and $\Delta_{P_{i},P_{i+1}}$ is the distance between two consecutive positions on the mobility model of the sink.

The sensor node deployment scenario for the protocols differs from one another other, and the deployment paradigm followed was based on the scenario discussed in the original paper. This was done because our intention was to compare the performance metrics between planned deployment and random deployment.

**Figure 7:**
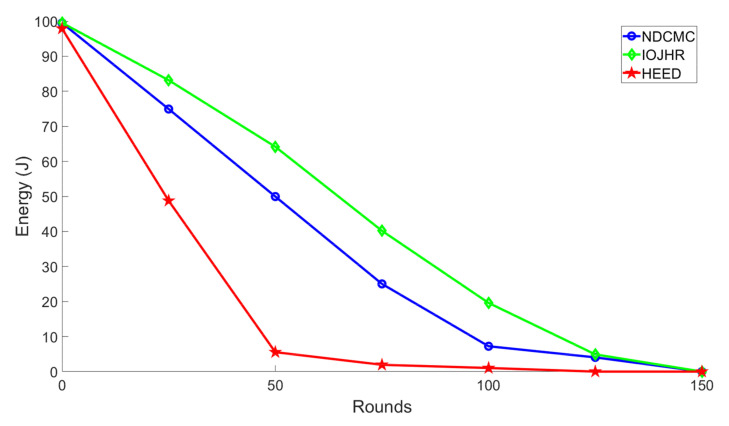
Analysis of energy performance.

Figure 7 illustrates the energy consumption scenario of the compared protocols. Based on the data shown, it can be seen that the IoJHR protocol performed better than the other two methods. This outcome was expected, as the IoJHR protocol follows a planned deployment, and the distance from the planned route of the sink is uniform. The CHs transmit data only when the sink is in close proximity. The main problem with NDCMC is that it does not optimize altitude with the sensor position. As a result, the transmission distance was comparatively higher with the IoJHR protocol. It should be noted that when the CH fails, the VHs and other sensor nodes are able to send data to the sink directly. Cluster range is an important factor in the energy consumption of the NDCMC protocol. If the cluster range is reduced, the intra-cluster distance is similarly reduced; consequently, the energy consumption due to data transmission will be lower. However, the amount of energy consumed by the HEED protocol was interesting. Based on the setup adopted in the simulation of this study, the HEED protocol exhibited a sink in the middle of the ROI. However, this may be due to a long transmission distance in the case of the CH, but not for the CMs, as the CHs are selected based on their lowest transmission power. The HEED protocol consumes more energy because it transmits the control packets for electing the CHs as distinct from the other protocols.

**Figure 8:**
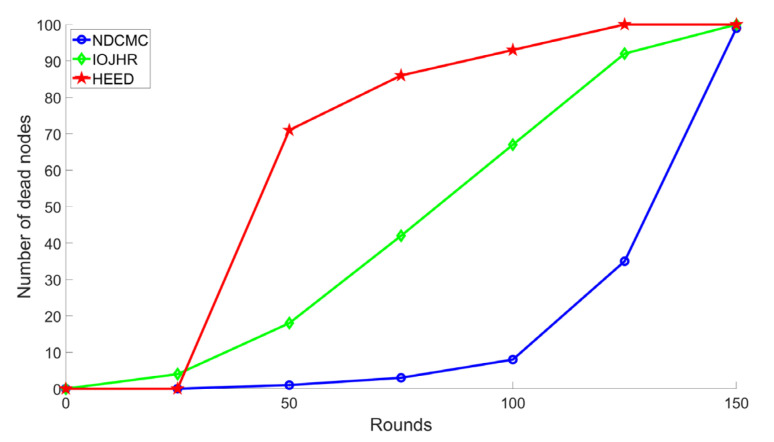
Analysis of sensor node lifetime.

Figure 8 shows an analysis of the number of dead nodes compared to the number of rounds. The terminology rounds state the dissemination procedure of a specific number of data packets from sensor point of view. The static sink-based solution simply disseminates the specific amount of data to the sink. However, in case of mobile sink-based solution, a round ends when the mobile sink completes a single round of data collection tour and collects the same number of data that is supposed to be disseminated in the static sink-based solution. In every round, the CH selection process is initiated. The graph clearly shows that NDCMC outperforms the other frameworks. According to the graph, the nodes in the IoJHR protocol die linearly. Among the compared protocols, nodes died significantly in the HEED protocol, and the energy of the majority node was reduced to zero before the 70th round. This situation occurred because of the transmission distance from the sensor nodes to the CH or from the CH to the sink. The IoJHR and HEED protocols cannot directly transmit data to the sink. However, with NDCMC, the transmission distances are smaller, and after the death of a CH, no further CH is selected, and the data are sent directly to the sink node. Therefore, the extra burden of the CH has little impact on NDCMC. The protocol also introduced VHs to solve the problem of long-distance messages from the CMs to the CHs. An expected outcome was to keep the nodes alive as long as possible, after which the sudden death of the majority of the nodes would occur over a given time. This outcome ensures a load-balancing data-collection mechanism in terms of energy consumption.

**Figure 9:**
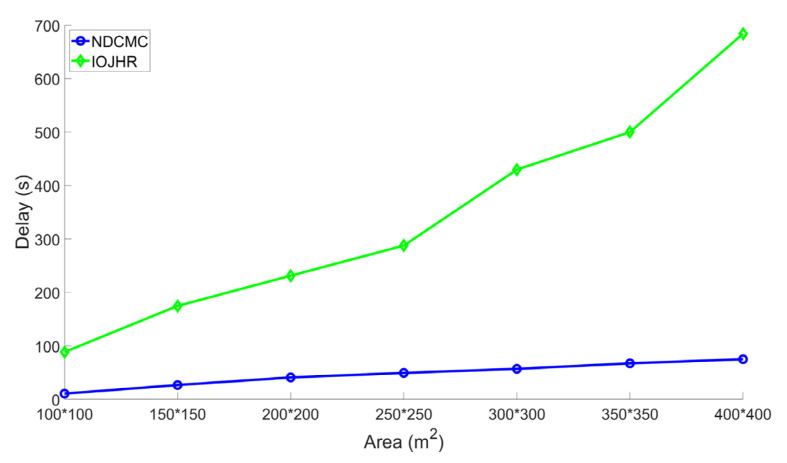
Analysis of delay performance.

To analyze the delay performance, we only considered the mobility of the sink nodes. The propagation delay is very low compared to the physical mobility of the sink. Two variables play a major role in the given delay analysis. The delay was compared to the area of the ROI. This figure also shows the delay-scalability performance of the adopted mobility model. From Figure 9, it can be seen that in the initial area size of 100 × 100 m^2^ to the last area size of 400 × 400 m^2^, NDCMC performed better than the IoJHR protocol. Not only did NDCMC perform better than the IoJHR protocol, but the delay also remained more or less consistent for NDCMC, whereas the delay exhibited a sharp increase with increasing area for the IoJHR protocol. This is because of the adopted sink node mobility models in the respective protocols. According to Figure 4, the IoJHR protocol follows a sink node mobility model, in which it moves back and forth in the ROI to collect data from the CHs. The mobility model ensures that data transmission occurs after reaching the optimal distance from a CH (but with increasing area size, the sink needs to travel an equal added distance). For NDCMC, the sink node follows a predefined mobility, where the stopping position is fixed by default. The mobility of the sink node in NDCMC is maintained using the greedy algorithm. It slightly increases with the increasing area of the ROI but does not entirely depend on this area. In Figure 6, the mobility model for NDCMC is explained. This is important because ground-based robot mobility is not easy to plan as it depends heavily on the type of terrain.

**Figure 10:**
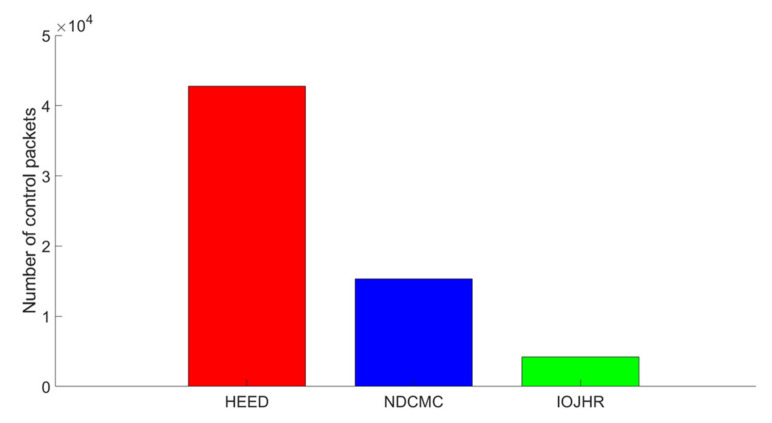
Analysis of control packet consumption.

Figure 10 illustrates the comparison of the control packet analysis among the investigated data-collection mechanisms. The data is collected by running the simulation five times, where each time consists of ten rounds. From the data, the average number of control packets consumed for a single round is depicted in the graph. Based on the theory and working mechanisms given in the original papers, the NDCMC and IoJHR protocols make use of GPS information. However, the HEED protocol does not require GPS assistance. The HEED clustering process consumes routing packets to form clusters and to select the CHs. NDCMC selects only the CH, and clustering is performed geographically. Mobile sink-based frameworks assume that the sensor nodes already know the predefined path of the mobile sinks. Another major assumption made by the IoJHR protocol is that the sensor node also knows the operating time and speed of the mobile sink so that the CHs can deliver data at the exact moment the mobile sink is in the vicinity. Although this is a drawback of the system, this mechanism helps to reduce the control packet consumption of the compared protocols. The NDCMC framework also utilizes GPS information. However, this framework receives beaconing assistance to alert the sensor nodes to start sending data. This criterion results in the consumption of more control packets. A cluster is formed in the BS, and the nodes do not need to form a cluster on their own, resulting in a smaller number of control packets. Conversely, in the HEED protocol, the mechanism does not require any help from GPS. Consequently, to form a cluster and the CH, many control packets need to be exchanged. As a result, the number of control packets in the HEED protocol is the largest among the investigated protocols. For these results, we used 30 rounds of data and then averaged the total number of control packets per round.

**Figure 11:**
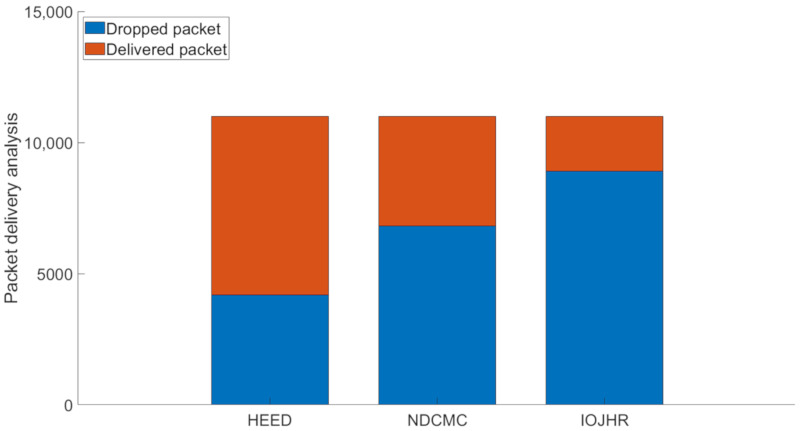
Analysis of packet delivery and drops.

Figure 11 shows the number of packet drop scenarios among the investigated WSN data-collection methods. It should be noted that the generated packet numbers for three of them were equal, as were the aggregation percentages. The data is collected from the first ten rounds of transmission for five times, and the average result for a single round is shown in the graph. The reason behind the high number of packet drops in the IoJHR protocol is its unsynchronized sink mobility, i.e., its sink path is predefined. The CHs calculate the presence of a sink based on predefined information. Any fluctuation in the sink timing will cause a bad result. NDCMC uses beaconing techniques to initiate data transmission, resulting in a lower number of packet drops compared to the IoJHR protocol. However, after the death of the CH, the nodes send data directly to the aerial sink, the transmission distance being higher which causes packet drops. As the nodes only have a small window for transmitting data, no speed optimization is performed based on the volume of transmission data. It should be noted that all the nodes have an equal transmission range; therefore, for the equal speed of a sink, the CHs will have an equal-sized window in which to transmit data. Thus, a small degree of asynchronization, with the higher volumes of data, results in poorer performance. For the HEED protocol, the sink is always present in the ROI, and the distance from the CHs is optimal. However, the CH-to-sink transmission distance makes for a long transmission time. This results in a lower number of packet drops.

## 5. Lessons Learned and Recommendation

This section consolidates the knowledge obtained from the simulation outputs and the theoretical information provided in the considered studies. At first, we list the lesson learned from the study as follows:

- Sink Mobility vs. Delay

The delay will increase significantly if the mobile agent does not follow any optimized trajectory. With an increasing area, if the delay is not addressed, the model will fail [71].

- Sink Mobility vs. Energy Consumption

Energy consumption increases if the sink mobility is not adaptive and does not change based on the CH position [72]. Pre-assuming the mobility of a sink is not valid, and it will lead to a high number of packet drops.

- Number of CHs vs. Energy Consumption

If the number of CHs is too small compared to the number of CMs, the amount of energy consumed by the CHs will be higher owing to the volume of received data [73]. Even though the energy consumption of receiving data is less than that of transmitting it, receiving a significant volume of data will consume considerable energy. Conversely, if the number of CMs is too high compared to the number of CHs, the volume of transmitted data will also increase, thus consuming more energy.

- Distributed Clustering vs. Control Packet Overhead

A distributed clustering algorithm increases the number of exchanged control packets to form clusters. By contrast, the static infrastructure-assisted clustering algorithm is performed by the infrastructure itself, resulting in a lower number of exchanged control packets [74].

- Geographical Knowledge vs. Control Packet Overhead

Prior knowledge of positional information creates extra pressure on buffer memory; however, the number of control packets is reduced. In the absence of GPS information, one way of gathering neighboring data is to estimate the distance of the neighboring node based on the received signal strength and angle of arrival. However, despite having flaws, the GPS is more accurate than the aforementioned mechanism. By contrast, the GPS does not provide good results in an indoor system, which may trigger other mechanisms. In such a scenario, the number of control packets can only be a secondary optimization parameter.

- Sink Mobility vs. Number of Packets Dropped

Sink mobility is directly related to the number of packets dropped. The sink speed can vary from very high to low—the sink may or may not have a variable speed for transmitting data to the CHs. For this reason, the CHs may not have enough time to transmit all of their data in a single round. Consequently, the data remains in the buffer of the CHs and may cause buffer overflows. In a mobile sink-based WSN data-collection protocol, none of the protocols have any kind of adaptive mobility for the sink’s mobility [75].

- CH Position vs. Data-Collection Position

In the investigated WSN data-collection mechanism, the CPDC protocol selects the CH based on a predefined sink position. This is a poor setup, as there is no mechanism adopted for the data-collection position to be adopted with any changed position of the CHs.

- Planned Deployment Reality vs. Expectation

The IoJHR protocol provides better results in terms of energy consumption and dead nodes compared to other protocols. However, the IoJHR protocol assumes that a node is the planned deployment. This is the biggest disadvantage of this approach. The clustering mechanism and CH selection mechanism are also heavily dependent on the planned deployment. Thus, in the case of random deployment and pattern less deployment of a real-life scenario, the IoJHR protocol will yield poorer results.

Based on the lessons learned, the recommendations for designing a well-balanced data-collection mechanism in WSNs are as follows:

- Adaptive Speed of the Sink

The mobile sink should exhibit adaptive speed by maintaining the amount of data to be transferred from a CH. The speed of a sink can reach up to 30 m/s. A CH might not have sufficient transmission windows to transfer all the data. However, if the sink is aware of the data to be transmitted and adjusts its moving speed accordingly, this limitation can be reduced. However, this also increases the volume of control packet consumption, which increases the packet delivery ratio. It is a serious problem to consider if all the data have equal priorities.

- CH Position-Based Data Collection and m-Mobility Adjustment

The CH selection mechanism depends on various parameters such as position, energy level, node degree, inter-cluster distance, and intra-cluster distance. If the sink adjusts its positions based on the position of the elected CHs, the transmission distance will be reduced, and the CHs will have a larger transmission window. As a result, the energy consumption will be significantly reduced compared to the energy consumption in a static infrastructure-based solution.

- Optimal Number of Clusters

From the simulations, we observed that in the case of energy consumption, CPDC performed the worst. One of the primary reasons behind this phenomenon is the smaller number of clusters with a high number of CMs. The CHs die early owing to their (receiving) energy consumption. If the number of clusters is too high and single-member clusters increase, the energy consumption due to CH-sink long-distance data transmission increases. It can be seen that these two contradictory situations create an optimization problem from which the optimal number of clusters can be derived.

- Multiple CHs or Introducing Different Role

An alternative solution is to have more than one CH in a cluster. A CH failure may occur at any time before the next round of data collection. This may result in the loss of a large volume of data. However, data duplication to a node with the role of an assistant CH may provide a solution.

- Data Propagation via Multi-hop CH Approach to the Sink

In the case of a large ROI and static sink, the CHs can propagate data in a multi-hop fashion. This helps energy consumption in two ways. First, the transmission distance is shorter; second, the data can be aggregated by propagating to the sink.

- Multi-Sink-Based Solution

Multi-sinks can be used for time-constrained data collection and to reduce energy consumption. In this way, a CH can deliver data to the nearest sink. As the sinks are not energy-constrained and the transmission range is considerably higher than that of sensor nodes, they can transmit the data to the desired destination.

- Priority Based Transmission

Prioritizing data has many benefits. In some cases, if a sensor node is about to die, it can only transmit prioritized data. For the mobile sink scenario, the less prioritized data can be transmitted at a later stage, when the sink gets closer to the CH.

## 6. Conclusions

In this paper, we have discussed the three categories of data-collection frameworks in WSNs based on sink type. The different types of sinks under study are static sink, ground mobile sink, and aerial mobile sink. The main objective of this study is to find out the suitable application scenario by discussing and finding the relative performance of the data collection frameworks in WSNs depending on the sink types. In order to do so, we have discussed and compared ten different data-gathering frameworks divided into three categories. The frameworks have been qualitatively analyzed in terms of their working principles, advantages, and limitations. Two tabular comparisons along with an elaborate analysis present the in-depth overview of the investigated protocols. The tables have been produced based on the main ideas, optimization criteria, and performance evaluation parameters. In addition, we have selected three representative frameworks from the investigated frameworks for quantitative comparison, where their performance has been evaluated in terms of energy, the number of dead nodes, delays, packet drop ratios, and the number of exchanged control packets. The lessons learned and recommendations have been provided as the outcome of our comparative study, which will assist future researchers in producing more suitable and versatile WSN data-gathering frameworks. After all the analysis, we can conclude that all the sink types have their own advantages and disadvantages based on the application areas. For a delay-constrained application scenario, a static sink is more suitable option. A ground robot sink is applicable to the accessible terrain, and the WSN’s lifetime can be elongated as well. However, for a hard-to-reach and infrastructure-less environment, an aerial sink is the only solution. Lastly, it can be inferred that a more robust solution is possible in terms of QoS and fault tolerance by introducing different types of sinks in a single solution and there is a gap in such research in the literature.

## Figures and Tables

**Table 1:** Summary of existing surveys on data gathering in WSNs.

Ref.	Main Contribution
[44] 2011	In this work, a thorough discussion is provided on the mobile element (ME) based data collection protocols in WSNs. Besides, the impact of an ME is provided, based on its trajectory, motion, and speed. This paper also presents a discussion on different forms of MEs practiced in the literature.
[51] 2013	In this work, eight data collection protocols in WSNs are critically analyzed, and the obtained results are shown in tabular format. The discussed schemes are EEDC, CAG, GSC, SBR, SCCS, DQEB, LEACH, and PEGASIS. Special discussion is given on multipath and hybrid data collection protocols.
[52] 2013	This work presents an in-depth discussion on the distributed database management systems proposed for WSNs. Alongside the advantages and disadvantages of data and query management techniques, an effective future research direction is also provided.
[53] 2020	This survey focuses on the routing mechanism of WSN-aided target tracking application by presenting the strengths and drawbacks of the existing schemes. A quantitative comparison is given based on energy consumption, data gathering efficiency, and target tracking time.
[54] 2014	In a time-constraint data collection scenario in WSNs, a real-time database management system can play a vital role. This paper provides a short description of such studies and proposes a model for simulation in a distributed environment.
[55] 2016	The research work consolidates data gathering techniques proposed from a different perspective such as networking, compressive sensing, signal processing, and information theory. The author provided an analytical model to obtain the energy performance and conducted the simulation to point out the best performing technique.

**Table 2:** Main ideas of the investigated WSN data-gathering frameworks.

Protocol	Ref.	Main Idea
TEEN	[56]	A *H_T_* and *S_T_* value are used by the sensor nodes for transmitting data to the CH that reduces the number of data transmissions
PEGASIS	[57]	A chain-based mechanism is used for one data transmission per round to the BS through data fusion among sensor nodes
HEED	[58]	A distributed clustering method is used that considers node residual energy and proximity for CH selection
CTP	[60]	A tree-based protocol that dynamically adapts the routing path based on the transmission cost
CPDC	[61]	A clustering method is used in which CHs are selected based on residual energy and distance, and a relay CH is selected that is closest to the sink
IoJHR	[62]	Several virtual grids are created with one CH in each grid which forwards data to the mobile sink when it is closest
EPMS	[63]	Clustering is performed by PSO and the CH is selected based on the maximum residual energy in each cluster
NDCMC	[64]	Clustering and CH selection is done based on node density; additionally, VH is also used for data gathering
UEWDC	[65]	A suboptimal solution is given for joint optimization of the UAV trajectory and node wake-up scheduling
UAVCDG	[66]	CDG is performed to reduce the volume of data transmission and the UAV trajectory is minimized

**Table 3:** Comparison of key optimization parameters of the investigated WSN frameworks.

Protocol	TEEN	PEGASIS	HEED	CTP	CPDC	IoJHR	EPMS	NDCMC	UEWDC	UAVCDG
Energy-saving technique	Rotation of CH	Rotation of the leader node	Considering intra-cluster communication cost	Adjusting beacon interval	Limited re-clustering	Limited number of hops	Keep selected CH on the next round	CH substitution by VH when out of power	Only one node wakes up at each time slot	CH selection to shorten the UAV trajectory
Data-gathering method	Hierarchical clustering	Chain construction with data fusion	Distributed clustering	Collection tree creation	Clustering and chain construction among CHs	Clustering	Clustering	Clustering and optimal track planning	UAV trajectory planning and node wake-up scheduling	Compressive data gathering and clustering
Node mobility	No	No	No	No	No	No	No	No	No	No
Location awareness	No	No	No	No	Yes	Yes	Yes	Yes	Yes	Yes
Sink energy constraint	No	No	No	No	No	No	No	No	No	Yes
Residual energy	No	No	Yes	No	Yes	Yes	Yes	Yes	No	No
Number of hops	No	No	No	Yes	No	Yes	No	No	No	No
Neighbor node proximity	No	Yes	Yes	Yes	Yes	No	No	Yes	No	Yes
Transmission distance	No	Yes	Yes	Yes	Yes	Yes	No	Yes	Yes	Yes
Energy efficiency	Yes	Yes	Yes	Yes	Yes	Yes	Yes	Yes	Yes	Yes
Network lifetime	Yes	Yes	Yes	No	Yes	Yes	Yes	Yes	No	Yes
Data-gathering latency	No	No	No	Yes	No	Yes	Yes	Yes	No	No
Packet delivery ratio	No	No	No	Yes	No	Yes	Yes	No	No	No
Number of dead nodes	No	No	No	No	No	Yes	No	Yes	No	No
Number of rounds	No	Yes	Yes	No	No	No	No	No	No	No
Number of nodes alive	Yes	No	No	No	Yes	No	Yes	Yes	No	No

**Table 4:** Simulation parameters.

Parameter	Value
Area	100 × 100 m^2^–400 × 400 m^2^
Number of sensor nodes	100
Number of packets sensed by a node in a single round	100
Initial energy	1 J
$E_{elec}$	50 nJ/bit
$\varepsilon_{fp}$	10 pJ/bit
$\varepsilon_{mw}$	0.0013 pJ/bit
$\varepsilon_{aggr}$	5 nJ/bit
Data packet length	4 KB
Hello packet length	100–150 B
Aggregation percentage	10%
Sensor mobility	Static
Carrier frequency	2.4 GHz
UAV altitude	50 m
Node deployment—IoJHR	Planned
Node deployment—NDCMC	Random
Node deployment—HEED	Random
Sink Placement—HEED	Center of the ROI
Antenna type	Omnidirectional
MAC protocol	CSMA, TDMA
